# New transcriptional-based insights into the pathogenesis of desmoplastic small round cell tumors (DSRCTs)

**DOI:** 10.18632/oncotarget.16477

**Published:** 2017-03-22

**Authors:** Tiziana Negri, Silvia Brich, Fabio Bozzi, Chiara V. Volpi, Ambra V. Gualeni, Silvia Stacchiotti, Loris De Cecco, Silvana Canevari, Annunziata Gloghini, Silvana Pilotti

**Affiliations:** ^1^ Department of Diagnostic Pathology and Laboratory Medicine, Laboratory of Experimental Molecular Pathology, Fondazione IRCCS Istituto Nazionale dei Tumori, Milan, Italy; ^2^ MOSE-DEA, University of Trieste, Trieste, Italy; ^3^ Department of Diagnostic Pathology and Laboratory Medicine, Fondazione IRCCS Istituto Nazionale dei Tumori, Milan, Italy; ^4^ Adult Mesenchymal Tumor and Rare Cancer Medical Oncology Unit, Cancer Medicine Department, Fondazione IRCCS Istituto Nazionale dei Tumori, Milan, Italy; ^5^ Department of Experimental Oncology and Molecular Medicine, Functional Genomics and Bioinformatics, Fondazione IRCCS Istituto Nazionale dei Tumori, Milan, Italy

**Keywords:** GEP, MErT/EMT, immunological ignorance, AR, Meflin/PDGFRA

## Abstract

To gain new insights into desmoplastic small round cell tumors (DSRCTs) by means of gene expression profiling (GEP). Formalin-fixed, paraffin-embedded surgical specimens obtained from seven pretreated DSRCT patients were interrogated using GEP complemented by immunohistochemistry, a cancer stem cell array, and miRNA *in situ* hybridisation, including the combined chimera modules miRNA-200/ZEB1 and miRNA-34/SLUG. The chimera modules divided the cases into three classes that respectively recapitulated the traits of mesenchymal epithelial reverse transition (MErT), epithelial mesenchymal transition (EMT), and hybrid/partial EMT. This indicates a close correlation between the reprogramming governed by EMT regulators and DSRCT biology, which was further confirmed by miRNA-21 and is consistent with the broad morphological spectrum of DSRCTs. Starting from the miRNA-200/ZEB1 axis, we also found that DSRCTs carry a signature of immunological ignorance that is not responsive to PD-L1 blockade. Evidence that the up-regulation of miRNA-200 and E-cadherin, and quite a high level of miRNA-21 expression segregate with the MErT supports the idea that, in addition to the hybrid/partial state, MErT is also enriched in stemness: the androgen-positive cases, whose stemness traits were confirmed by stem cell arrays, all fell into these two classes. Our findings also confirmed that tumoral cell PDGFRA expression correlates with desmoplasia, and demonstrated the co-expression of PDGFRA and ISLR/Meflin, another marker of pluripotency. Despite the limited number of cases, these findings provide unexpectedly relevant information concerning the pathogenesis of DSRCTs, and prove the validity of miRNA-based chimera circuit modelling in the clinico-pathological setting.

## INTRODUCTION

DSRCTs are rare, ominous, small round cell sarcomas mainly affecting young men that bear the *EWS-WT1* translocation and are therefore included with simple sarcomas in the molecular sarcoma classification [1]. It has been shown that the *EWS-WT1* chimera leads to the transcription of a number of the target genes involved in proliferation, survival and invasion, including *PDGFRA* [2]. Furthermore, it has been reported that a number of fusion transcription variants (including full-length WT1) may give rise to an atypical staining pattern using antibody against the C terminal of WT1 [3] that may complicate an immunophenotypical diagnosis. Somatic *MET* and *PIK3CA* mutations have been identified [4], and there have been reports of the deregulation of epigenetic modifications, such as the expression of lysine specific demethylase (LSD1) [5], and the loss of INI [6] and SMARCA4 [7], thus suggesting that the SWI/SNF chromatin remodelling complex may play a role in DSRCTs. In an attempt to gain more insights into the molecular alterations of this rare disease, which is locoregionally disseminated at the time of diagnosis in the overwhelming majority of cases and therefore is expected to be medically treated before surgery, we used gene expression profiling (GEP) in order to find clues concerning possible pathways of interest. Among the biological pathways associated with the two identified clusters, we considered epithelial mesenchymal transition (EMT), androgen receptor (AR) in addition to a gene associated with PDGFRA. EMT and the mesenchymal epithelial reverse transition (MErT) are complex processes involved in normal development and diseases such as cancer and fibrosis that are driven by EMT regulators (SLUG, ZEB1, TWIST and others) and miRNAs together with epigenetic regulators [8]. The concept of partial EMT has recently been introduced into the field of EMT/MErT research [9] and, on the basis of this new view, EMT is no longer regarded as “all or nothing” process, but as a process in which the cells move through a spectrum of intermediate phases involving partial EMT. Partial EMT is also referred to as a hybrid and metastable state, definitions that highlight the ability of these cells to co-express epithelial and mesenchymal markers, and thus induce or revert EMT [10]. This hybrid state is governed by modules consisting of miRNA-transcription factor (TF) pairs called “translational-transcriptional chimera” [11] or “chimeric modules” [12], which serve to determine the three phenotypes of MErT, hybrid/partial EMT, and full EMT. Herein, GEP, performed on formalin-fixed material, complemented by immunophenotyping, cancer stem cell array, and *in situ* hybridisation (ISH) analyses provided a comprehensive picture of the pathogenetic alterations characterising DSRCTs, which can be summarised in the following four main cues. First, a high degree of plasticity due to the metastable state of hybrid/partial EMT tumor cells that at one hand favours tumor stemness and local spreading, and on the other hand is consistent with the broad morphological spectrum of the disease. Second, a PD-L1 immunoscore of both tumor cells and host immunocomponent that fits with the “non-inflamed” tumor type. Third, an unexpected enrichment in the stemness of AR-positive cases that may explain the limited effectiveness of this target treatment in DSRCTs. Fourth, a reinterpretation of the role of PDGFRA which, together with ISLR/Meflin (a new player closely related to pluripotency), is confirmed as contributing to desmoplasia.

## RESULTS

### GEP analysis

**Figure 1:**
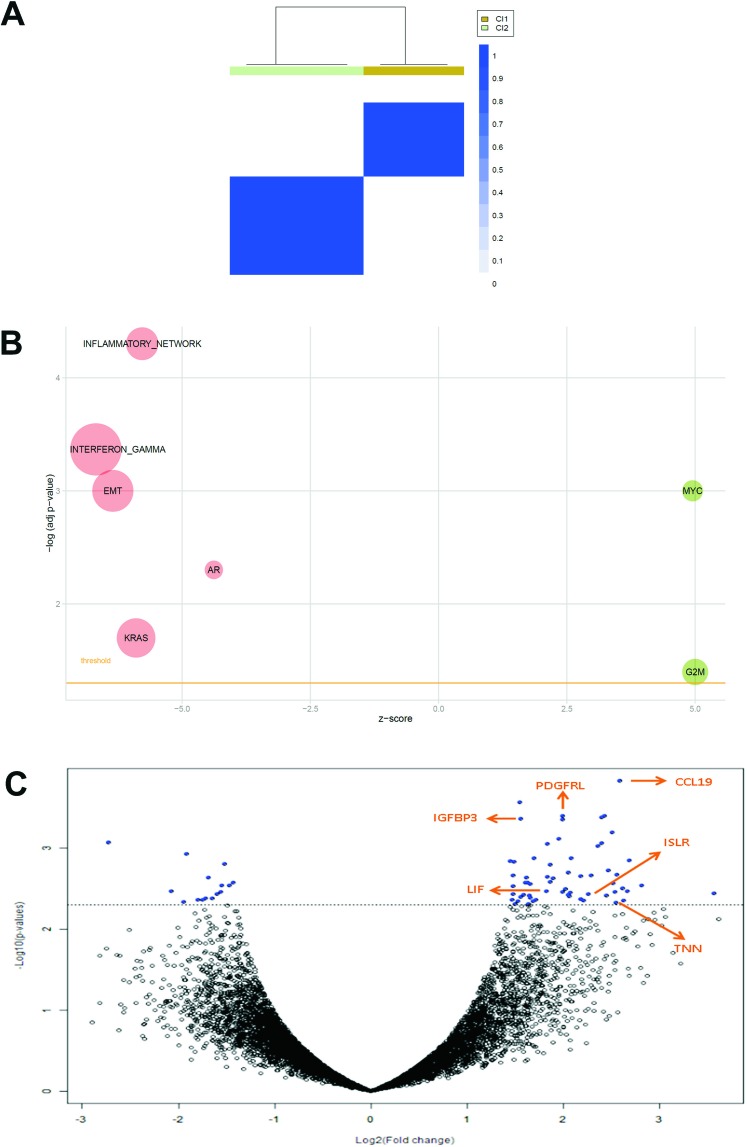
**A**. Consensus unsupervised clustering analysis. The heatmap depicts the consensus matrix imposing the presence of two clusters on the dataset. The values range from 0 (white, samples do not cluster together) to 1 (blue, samples showing high clustering affinity). **B**. Bubble plot of gene sets associated to DSRCT sample clusters. An overview of GSEA enriched networks was depicted through bubble plots. The x-axis indicates the z-score for each term, while the y-axis is the negative logarithm of the adjusted p-value. The area of the displayed circles is proportional to the number of genes assigned to each term. Green = pathways enriched in Cluster 1; Red = pathways enriched in Cluster 2. **C**. Volcano plot of log2 (fold changes) versus -log10 p-value. The volcano plot shows transcriptional differences between the two groups of samples identified by consensus unsupervised clustering analysis. The horizontal dashed line denotes the FDR<0.15 cut-off. Arrows indicate six pivotal genes.

Consensus unsupervised clustering was applied to the DSRCT expression data matrix. Taking into account the most variant genes, the clustering analysis revealed two main groups of samples, and the consensus heatmap provided evidence that these appeared to be well defined (Figure 1A). The biological pathways associated with the two clusters were investigated using the gene set enrichment analysis (GSEA). As shown in Figure 1B, cluster 1 was enriched in the MYC pathway and G2M cell cycle, and cluster 2 was markedly enriched in a number of pathways, including the interferon gamma and inflammatory responses, EMT, AR and KRAS. We also investigated the differentially expressed genes, and we identified 77 genes by class comparison: 62 up-regulated in cluster 1, and 15 up-regulated in cluster 2 (Figure 1C, Supplementary Table 1). We decided to focus on the EMT and AR pathways, as well as *ISLR/Meflin*, a gene that was significantly differentially expressed in cluster 1 (Supplementary Tables 1 and 5). As all of our cases had been pre-treated, we choose not to investigate the interferon gamma and inflammatory response pathways in detail because it is acknowledged that chemotherapy may trigger innate activation and adaptative T cell responses [13, 14] furthermore, it has also been noted that effector T cell related chemokines may be produced by tumor cells and secreted by stromal cells [15]. Nonetheless we performed, an immunoscore assessment of the immune system-based biomarkers including CD3, CD20, CD14 and CD163 which showed that, with the exception of focal immunoreactivity for CD3 in one case (see below), the samples were negative for the first two markers, and revealed scattered clusters of CD14 and CD163 restricted to the tumor stroma in three cases (DSRCT2, DSRCT4, DSRCT6 cases, Supplementary Table 2). The absence of an inflamed T-cell phenotype indicates a DSRCT phenotype corresponding to immunosystem exclusion or ignorance with a myeloid (albeit low) component [16].

### MErT/EMT govern the considerable plasticity of DSRCTs

**Figure 2:**
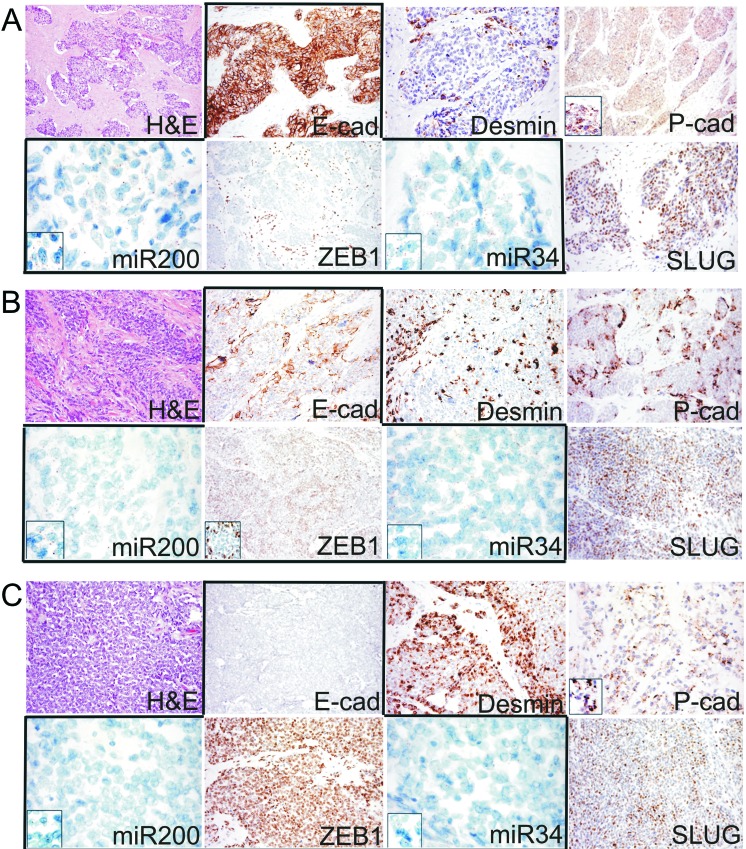
**A**. Example of the epithelial phenotype correlating with MErT, that was characterized by high miR200/miR34 and null ZEB1 expression coupled with strong E-cadherin immunoreactivity (group 1, DSRCT 2). **B**. Example of the hybrid/partial EMT cases that were characterised by the intermediate expression of both miRNAs and the heterogeneous expression of either ZEB1 or E-cadherin (group 2, DSRCT 5). **C**. Example of the EMT cases that were characterised by strong ZEB1, very low miR200/miR34, and null E-cadherin expression (group 3, DSRCT 7). The expression of SLUG was similar in all three states; desmin increased from MErT to EMT; P- cadherin was expressed in all three states, but its expression was increased in the hybrid/partial or metastable state, in which it outlined the membrane areas bounding the tumor nests. miR200/miR34: miRNA-200/miRNA-34.

**Figure 3:**
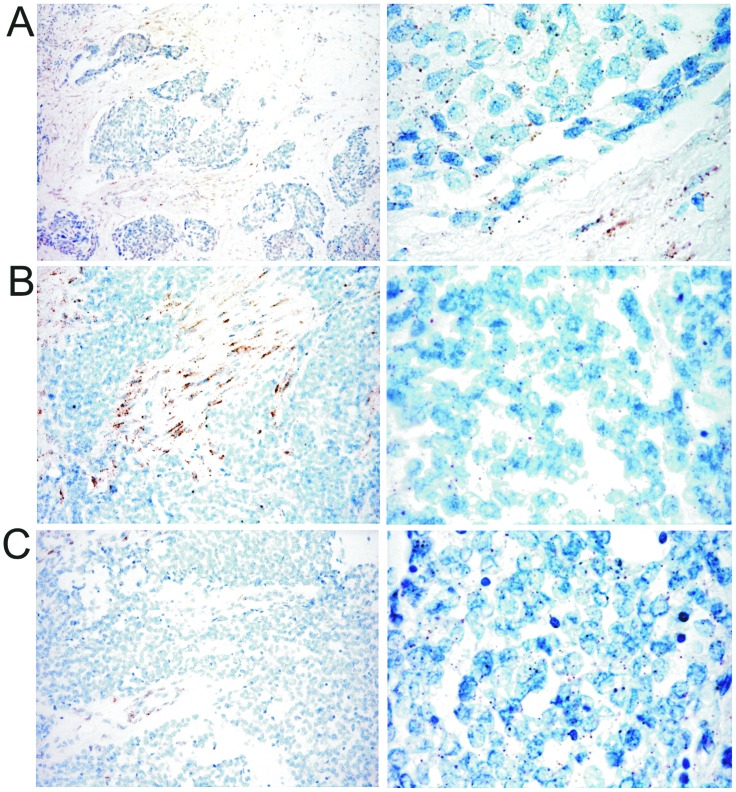
Example of the miRNA-21 signature in one case from each group (original magnification 200 X left column, 400 X right column) **A**. group 1 (DSRCT 2); **B**. group 2 (DSRCT 5); **C**. group 3 (DSRCT 7). The tumor cell expression of miRNA-21 was similar in the three groups and was enriched in signals in group 1 (A), in which it was largely expressed in stroma cells.

The GSEA indicated EMT pathway enrichment in cluster 2, and the differential expression analysis highlighted the up-regulation of three genes whose activation loops/signalling pathways are known to promote EMT in cluster 1: *CCL19, IGFBP3,* and *LIF* [17–19] (see arrows in Figure 1C). Despite the apparently conflicting results of the molecular analyses, we could not help noticing that both findings indicate the involvement of the highly plastic EMT/MErT process in DSRCTs. In order to investigate the MErT/EMT pathways further in an attempt to validate the GEP results, we used the recently proposed core regulatory system that makes it possible to predict dynamic gene expression during partial and complete EMT by demonstrating the existence of immunohistochemistry (IHC)/ISH based chimeric modules [12]. This system consists of modules of TF-TF or miRNA-TF pairs (the latter is also known as the chimera module) that can regulate decisions concerning the fate of cancer cells, and it is expected that the cancer cell state corresponds to the result of a mutually inhibitory pair of TF-TF or miRNA-TF genes, in which one or both genes are self activating, thus respectively leading to asymmetric or symmetric activation. The chimeric modules considered were miRNA-200/ZEB1 and miRNA-34/SLUG, both of which are associated with MErT/EMT: high miRNA-200/low ZEB1 and/or high miRNA-34/low-ZEB1 expression corresponds to an epithelial phenotype, whereas high ZEB1/low miRNA-200 and/or high ZEB1/ low miRNA-34 expression corresponds to a mesenchymal phenotype. The two chimeric modules were complemented by an immunohistochemical analysis which, in addition to ZEB1 and SLUG, included E-cadherin as a marker of an epithelial/MErT phenotype that is acknowledged to have important pluripotency and reprogramming functions [20], desmin as a marker of a mesenchymal/EMT phenotype, and P-cadherin, a recently proposed marker of hybrid/partial EMT and cancer stem cells (CSCs) [21]. On the basis of the chimeric module miRNA-200/ZEB1(see image gallery in Figure 2 and Supplementary Table 3), the cases were divided into three groups that were not clearly associated with GEP clusters: two cases recapitulating a MErT phenotype characterised by null ZEB1, high miRNA-200 and high E-cadherin expression (group 1); three cases corresponding to hybrid/partial EMT, all of which were intermediately positive for ZEB1, miRNA-200 and E-cadherin (group 2); and two cases consistent with EMT and characterised by high ZEB1, low miRNA-200, and null E-cadherin (group 3). The readout of the miRNA-34/SLUG module highlighted a trend towards the parallelism of miRNA-34 and miRNA-200, and the intermediate expression of SLUG in across all but one case, in line with its monostable state and its role as a noise-buffering integrator [12]. Desmin was patchily expressed in all of the cases, with immunodecoration going from MErT to EMT. Finally, P-cadherin was also mainly patchily expressed in all of the cases, but a pattern of immunolabelling outlining the boundaries of tumor nests was often observed in the hybrid/partial EMT cases (group 2, Figure 2), and there was diffuse salt-and-pepper decoration in MErT cases (group 1, Figure 2). We also focused on LIF, a multi-functional cytokine belonging to the IL-6 super-family [22] that is closely related to pluripotency [23] and has recently been associated with EMT [19], and that resulted differentially expressed by GEP. As LIF is a secreted protein whose very short half-life is due to serum protease [22], we chose to validate the transcriptome readouts by means of miRNA-21 ISH [19]. The analysis demonstrated the presence of signals in either tumor cells or the SMA-positive myofibroblasts present in the stromal component, a finding that is in line with the fact that, in addition to acting on tumoral cells, LIF also promotes the activation of cancer associated fibroblasts (CAFs) [24]. It is worth noting that, as shown in Figure 3, there was a similar number of signals in the three classes, which supports the idea that MErT is also enriched in CSCs. The discrepancy between our ISH and GEP results may be due to the fact that the extractive method melts tumoral cells and the stromal component (Figure 3). Taken together, the above findings refine the GEP results and are in line with the broad spectrum of morphological patterns found in this sarcoma. The prevalence of hybrid/partial EMT/MErT cases (five out of seven) enriched in CSCs (P-cadherin-positive) which are known to colonise or be primed for metastatic spreading [9], also give a new biological meaning to MErT, which should no longer be viewed simply as the expression of a more differentiated state, but also as an epithelial featuring carcinoma-like expression of cells enriched in stemness traits.

### EMT is not related to PD-L1 immuno-escape in DSRCTs

**Figure 4:**
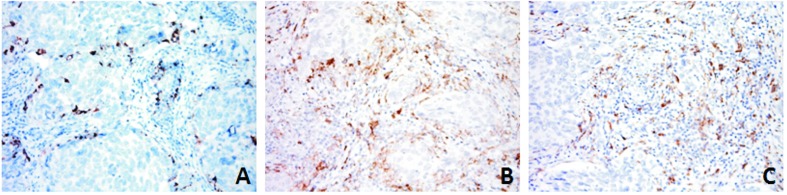
PD-L1 ( **A**) expression was restricted to the immune component and mainly decorated myeloid-derived cells, as shown by CD14 (**B**) and CD163 (**C**) immunostaining in DSRCT 2 case.

As it has been shown that ZEB1/miRNA-200 modulates the level of PD-L1, which suggests that the more mesenchymal-like tumor cells are intrinsically capable of immuno-escape [25], we extended the immunophenotyping of the immune host compartment (Supplementary Table 2) to PD-L1. None of the cases showed PD-L1 immunostaining of tumoral DSRCT cells, a finding that is in line with those of other authors [26] and indicates a tight histotype-related and context-dependent immune response [27]. Immune host component PD-L1 labelling was only observed in a single case, which was also the only one of the entire series showing an inflammatory infiltrate positive for CD3 upon H&E staining. In this case, the PD-L1 decoration involved only a small CD3 positive area (Supplementary Figure 1) that seemed to be enriched in CD14- and CD163-positive cells, which are known to contribute to the formation of a suppressive milieu and thus favour tumor escape (Figure 4). All of the other cases were PD-L1 negative. These findings reconfirm that the phenotypical signature of DSRCTs is one of “immunological ignorance”, which requires a distinct form of immunotherapeutic intervention [16].

### AR-positive cases segregate with the MErT-hybrid/partial EMT classes

**Figure 5:**
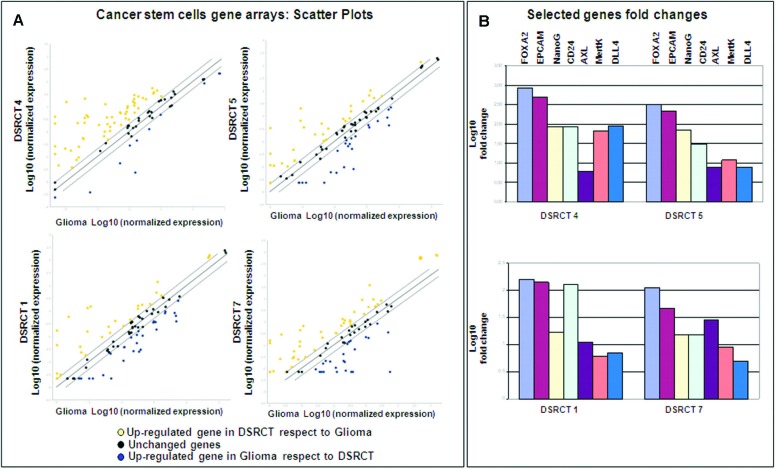
**A**. Scatter plot comparing the normalised expression of every gene between the selected DSRCT FFPE sample (Y axis) and the glioblastoma FFPE sample (X axis). The central line indicates unchanged genes. The dotted lines indicate the selected fold regulation threshold. The yellow and blue dots respectively indicate the genes in the DSRCTs that are up- and downregulated in comparison with the glioblastoma; the black dots indicate unchanged genes. **B**. Foldchanges were calculated using the 2 ^−ΔΔ Ct^ method with the glioblastoma sample as a reference, and are expressed as Log10.

The GEP analysis indicated that the AR gene set was enriched in cluster 2. AR is a pivotal player in this network and was found to be significantly differentially expressed between the two clusters. For purposes of validation, we made an IHC analysis that extended the immunoprofile to AR and β-catenin (one of the five uppermost co-activators induced by AR) [28], and clearly showed that AR-negative cases tended to segregate with cluster 1 (two out of three) and AR-positive cases with cluster 2 (three out of four) (Supplementary Table 4). However, when we divided the cases on the basis of the chimera module, we found that the AR-positive cases segregated with the MErT and hybrid/partial EMT classes (four out of five) (Supplementary Table 4 last column). The IHC analysis also highlighted an overlap between the AR and β-catenin profiles (Supplementary Figure 2) and there was no evidence of an inverse correlation between AR activation and E-cadherin down-regulation. The fact that the AR-positive cases fell into the MErT-hybrid/partial EMT classes is consistent with their morphological and immunophenotypical signature, and indicates their CSC status. In order to confirm the enriched stemness, we used CSC arrays to compare the level of CSC gene expression of one MErT, two hybrid/partial EMT and one EMT DSRCT with that of one glioblastoma (a tumor known to harbour a high percentage of CSCs) [29]. The results showed the transcriptional enrichment of FOXA2 (an early marker of mesodermal commitment) [30], EPCAM and CD24 (markers of digestive tract CSCs) [31], NanoG (known to be involved in controlling pluripotency) [32], genes known to be over-expressed in mesenchymal stem cells (Axl and Mertk) [33] (Figure 5).

### ISLR/Meflin together PDGFRA segregates with desmoplastic component

**Figure 6:**
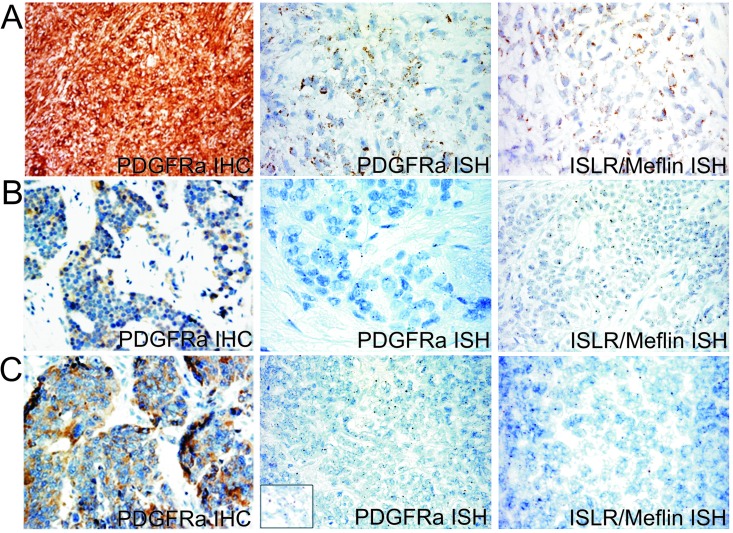
A PDGFRA mutated GIST (PDGFRA D842V, exon 8) used as a control showing intense PDGFRA immunostaining and strong granular cytoplasm signals of both *PDGFRA* and *ISRL/Meflin* by means of ISH A., heterogeneous and faint PDGFRA immunoreactivity and weak and dot-like mRNA *PDGFRA* and *ISRL/Meflin* signals in one group 1 case (DSRCT 4) B., and one group 2 case (DSRCT 5) C

The GEP over-expression of TNN, PDGFRL and ISLR/Meflin in cluster 1 (see arrows in Figure 1C) indicates that stromal fibrosis plays a role in DSRCTs. We investigated the expression of ISLR/Meflin, a pluripotency-related protein whose function is little understood [34] that has recently been linked to PDGFRA. In order to verify the occurrence of PDGFRA expression in our series and assess the possible mRNA expression of *ISLR/Meflin* and *PDGFRA*, we used PDGFRA immunostaining and, in the absence of a commercially available Meflin antibody working on formalin fixed paraffin embedded (FFPE) tissue, carried out ISH against *PDGFRA* and *ISLR/Meflin* using a PDGFRA-positive gastrointestinal stromal tumor (GIST) as an in-built control (Figure 6).

Two of the seven cases (both with a desmoplastic component) showed a low level of PDGFRA expression and ISH revealed the expression of *PDGFRA* and *ISLR/Meflin* mRNA; the remaining five were negative to either analysis. The mRNA expression was restricted to tumoral cells, as previously reported [34], but the *ISLR/Meflin* signals outnumbered those of *PDGFRA* in only one case (Figure 6). Altogether, the results confirm the link between desmoplasia and PDGFRA expression and add further stem-like traits to DSRCTs by demonstrating the previously unanticipated expression of ISLR/Meflin, which particularly seems to characterise the MErT class of DSRCTs.

## DISCUSSION

In this study, GEP was used as a starting point to identify candidate oncogenic pathways in DSRCTs, after which we used IHC, ISH and CSC array analyses in an attempt to contextualise and, when possible, provide a picture of the most significant processes dictated by the molecular analysis. The results demonstrated the high degree of plasticity/flexibility of DSRCT cells orchestrated by the EMT/MErT process, which explains the broad histological spectrum of the tumors. The relevance of EMT/MErT in this tumor is underlined by its dual epigenetic regulation via miRNA200/miRNA-34 and miRNA-21: the first is mainly involved in governing the switch, and the second endows pluripotency. Current thinking concerning “partial EMT” suggests that the metastable status of tumoral cells (i.e. the capacity of epithelial tumoral cells to undergo EMT and revert to an epithelial identity) is the key to the apparent paradox that the local progression of a primary tumor is facilitated by the acquisition of mesenchymal traits, whereas colonisation in metastatic sites requires rapid reversion to an epithelial phenotype [9]. The process of colonisation is fundamental when considering DSRCTs because of their characteristic tendency to arise from the abdominal or pelvic cavity as diffuse masses.

Using chimeric modules consisting of TFs (ZEB1 and SLUG) and miRNAs (miRNA-200 and miRNA-34), our DSRCTs could be divided into three groups that respectively recapitulated the MErT, hybrid/partial EMT and EMT phenotypes (Supplementary Table 3, Figure 2). In line with experimental data [12], the miRNA-200/ZEB1 double negative feedback loop together with the input from miRNA-34/SLUG restricted to miRNA-34 dissected the three classes. The new resetting suggested that most of the cases fell into the first two classes, which had a signature consistent with hybrid/partial EMT and MErT that closely recapitulated that observed during metastatic colonisation, and were enriched in E-cadherin and AR-positive cases. These findings are in line with the reported ability of E-cadherin to replace OCT4 (one of the four OSMK TFs) in driving and maintaining an undifferentiated/CSC state [35], and reinforce the idea of possible cross-talk between adhesion-mediated pluripotent states and TF-driven nuclear events in the stem cell reprogramming process. The link with stemness was strengthened by the IHC expression of P-cadherin and, indirectly, by evidence of the expression of miRNA-21 as a surrogate of LIF, a cytokine whose characteristics are close to both pluripotent cells and CSCs [23]. LIF is one of the genes expressed in unrestricted somatic stem cells, which have been described as recapitulating the characteristics of mesenchymal stem cells (MSCs) from placental cord blood and, among the recently proposed transcriptome-based subtypes of MSCs [23], are therefore the most closely related to human embryonic stem cells (hESCs). Accordingly, LIF (alone or in combination with bFGF), is the main factor maintaining the pluripotency of hESCs during derivation and long-term culture [36]. It has more recently been demonstrated that LIF induces the expression of miRNA-21, a miRNA that promotes EMT through STAT3 signalling in human tumor cells [19] and was shown in our ISH experiments to be expressed at similar levels in all three classes. In short, instability (the switch from epithelial to mesenchymal), heterogeneity (a mix of cells with more or less CSC-like characteristics), and the propensity to colonise all seem to be unfavourable factors characterising DSRCTs, and may explain the limited success of the various therapeutic regimens suggested so far. Furthermore, although there is pre-clinical evidence that ZEB1 induces PD-L1 expression in tumor cells by relieving miRNA-200 suppression (thus suggesting that the EMT phenotype may respond to checkpoint inhibitors) [25], the lack of PD-L1-positive DSRCT cells suggests that this is unlikely to have any therapeutic significance, although it goes without saying that the data must be confirmed in large case series. The segregation of AR-positive cases to the MErT and hybrid/partial EMT classes underlines a previously unreported discrepancy between the degree of differentiation (in terms of morphology and immunophenotype) and stemness, which may explain the poor response of DSRCTs to target treatments [37]. The absence of the nuclear co-localisation of AR and β-catenin despite the overlapping between the AR and β-catenin profiles is likely to be due to the HNI-mediated stabilisation of β-catenin and its interaction with E-cadherin [38,39]. Furthermore, unlike others [40] we did not observe an inverse correlation between AR activation and E-cadherin downregulation. Our results seem to be more in line with the hypothesis of the existence of an AR induced EMT in which, by by-passing the effects of canonical EMT activators [41], the high level of AR expression prevents a reduction in E-cadherin facilitating the hybrid state and the retention of stemness, findings that were confirmed by the results of our CSC array analyses. The possible existence of an AR-mediated EMT is a fascinating hypothesis, but it must be remembered that the AR axis may regulate EMT in unexpected and divergent manners [41] by exploiting different epigenetic regulators that, in addition to miRNAs, may involve nuclear receptor co-repressors (NCoRs), LSD1, Polycomb and SWI/SNF complexes, as suggested by our preliminary IHC analysis. We intend to investigate this in more depth by means of whole exome sequencing. Finally, among the three matricial proteins identified by our GEP analysis, we focused on ISLR/Meflin, a protein that has recently been reported to interact with PDGFRA, a marker that is historically related to the desmoplasia characterising DSRCTs [42]. ISLR/Meflin is a newly discovered surface and secreted pluripotency-related protein [23] whose over-expression has been described in cancer stroma [43] and fibrotic diseases, and a recent immunoprecipitation study has demonstrated its interaction with receptor tyrosine kinases (RTKs) such as EGFR and PDGFRA [34]. Intriguingly, after the finding of a positive correlation between PDGFRA tumor cell expression and stroma desmoplasia in a first study based on an *in vitro* analysis and IHC and ISH investigations of histological sections [42], a second and more recent IHC study has found that PDGFRA inversely correlates with tumor- associated desmoplasia [44]. In addition to confirming the data from the first study [42], our PDGFRA results underline the fact that this is a rare event and demonstrate that, together with PDGFRA, DSRCT cells express ISLR/Meflin which, once again, is pluripotency related [23]. Cumulatively, our findings indicate that DSRCTs are characterised by a high degree of plasticity that is governed by chimeric modules and miRNA-21 within the MErT/EMT process, in which MErT also seems to be enriched in stemness. It can be argued that DSRCTs are themselves multiphenotypical tumors expressing epithelial and mesenchymal traits, but the significance and magnitude of the modulation of epithelial and mesenchymal marker levels orchestrated by adhesion molecules (E-cadherin and P-cadherin), TFs (ZEB1 and SLUG) and miRNAs found in this study are qualitatively dramatically different as they involve pluripotency, differentiation and migration. Furthermore, despite the ability of the miRNA-200/ZEB1 axis to regulate both EMT and PD-L1 expression in tumor cells [25], our findings strongly suggest that DSRCTs fall into the subgroup of “non-inflamed tumor types” that are closely associated with MErT/EMT and stem-like-traits [45]. Finally, in the context of a profile of terminally differentiated cells, AR-positive cells harbour stem cell properties to which matricial-related ISLR/Meflin also seems to contribute. We are well aware that our series is too small to allow any definite conclusions, but we believe that these preliminary results may be helpful for future investigations. Furthermore, although it can be argued that our samples were obtained from treated patients and that some of the observed changes may be secondary, it is worth noting that our findings strictly depend on tumoral machinery and thus remain informative.

## MATERIALS AND METHODS

The study was approved by the Independent Ethics Committee of the Fondazione IRCCS Istituto Nazionale dei Tumori di Milano (INT-MI). All of the patients whose biological samples were included in the study gave their signed consent to donate the tissue remaining after the diagnostic procedure had been completed to INT-MI. We interrogated the database of the Department of Pathology at our Institution and identified seven primary DSRCTs surgically removed after combined chemotherapy between 2000 and 2016. The clinical data (including follow-up data when available) were obtained from the patients’ records and are summarised in Supplementary Table 5. The archived hematoxylin and eosin (H&E)-stained slides and IHC slides stained using a canonical diagnostic panel were retrieved and reviewed. In all cases, the presence of the *EWS-WT1* translocation was confirmed by means of fluorescent *in situ* hybridisation (FISH): a commercial probe KIT (Vysis EWSR1 Break Apart FISH Probe Kit) for EWSR1 break apart and an in house labeled BAC probe mix (RP11-259N9, RP11-299P16) for WT1 break apart. The antibodies and experimental conditions used for IHC staining in this investigation are detailed in Supplementary Methods.

### Whole genome gene expression profiling

The gene expression analysis was made using FFPE material obtained by direct dissection from ten 7 μm methylene blue-stained sections of non-necrotic, contamination-free normal tissue representative of tumoral areas. RNA was isolated using the Qiagen RNeasy FFPE kit (Qiagen, Valencia, CA, USA) in accordance with the manufacturer's instructions. The gene expression profiles were generated using Illumina HumanHT12_v4 WGDASL (cDNA-mediated annealing, selection, extension, and ligation) (Illumina, San Diego, CA, USA), which allows the expression profiling of 29,377 probes from degraded RNA. Reverse transcription, oligo annealing, ligation, amplification, labelling, probe purification, hybridisation, and chip washing procedures were carried out in accordance with the manufacturer's instructions. The Illumina IScan Reader was used to scan the arrays, and Illumina BeadScan software was used to acquire the images and recover the primary data, which were then normalised using BeadStudio software and the quantile method. The data have been deposited in the Gene Expression Omnibus repository (Accession No. GSE90904). The DSRCTs underwent unsupervised tumor subtype identification using k-means clustering and 1Pearson correlations as the distance matrix following the procedures implemented in the R package ConsensusClusterPlus [46] on the basis of 1000 re-sampling interactions randomly selected from a fraction of the samples. Given the number of samples, we tested the existence of k=2 clusters. The pathway analysis was made using gene set enrichment analysis (GSEA) [47] of a total of 556 gene sets (GS) with a false discovery rate (FDR) [48] of <0.15 that were identified on the basis of the GO terms. The degree of GS enrichment in the two identified tumor subtypes was expressed using a normalised enrichment score (NES); a NES with an FDR of <0.05 and |>1.5| was considered statistically significant. The most significant GS were graphically represented using the GOBubble function available in the GOplot R package [49], which displays information concerning the significance of the enrichment (the -log10 p-value) and the z-score of each GS. Differential expression was analysed using a random variance t-test that improved the estimates without assuming that all of the genes had the same variance. An FDR correction was applied to the raw p-values using a threshold FDR of <0.15. BRB-ArrayTools (version 4.3.1, developed by Dr. Richard Simon and the BRB-ArrayTools Development Team), which is available at http://linus.nci.nih.gov/ was used for the differential expression analysis.

### RNA *in situ* hybridisation (ISH)

MiRNA ISH was performed as previously described [50]. FFPE tissue sections were hybridised with double-DIG-LNA probes for *miR-21* (# 38102-15), *miR-34a* (# 38487-15) and *miR-200c* (# 38536-15); the negative control was the Scramble miR probe (# 99004-15) (Exiqon, Vedbaek, Denmark).

*ISLR/Meflin* and *pdgfra* mRNA expression was studied by means of ISH using the target probes ISLR (# 455481 and # 604481, Advanced Cell Diagnostics, Inc., Hayward, CA) and the RNAscope 2.5 High-Definition detection kit (brown) (Advanced Cell Diagnostics, Inc.) in accordance with the manufacturer's instructions (see Supplementary Methods for details).

### Cancer stem cell gene array

RNA was extracted as above described from one MErT, two hybrid/partial EMT and one EMT case, and 1 μg was retro-transcribed using the RT^2^ first strand kit (Qiagen, Cat. No. 330401). The RNA obtained from a representative FFPE glioblastoma sample obtained from an additional patient was included as a reference/control. After retro-transcription, the cDNA was pre-amplified using the human cancer stem cell RT^2^ PreAMP pathway primer mix (Qiagen, Cat. No. 330241 PBH-176Z) in accordance with the manufacturer's instructions. The pre-amplified cDNA was then used as a template for the human cancer stem cell RT^2^ profiler PCR array (Qiagen, Cat. No.330231 PAHS176ZA). The arrays were amplified using an ABI 7900 HT fast real-time PCR with RT^2^ SYBR Green ROX qPCR as the mastermix (Qiagen, Cat. No. 330520). All of the raw data (listed in Supplementary Table 6) were obtained using a value of 0.05 as a cycle threshold (Ct) and analysed using the bio-informatic tools available (personal registration is required) at the Qiagen web site (http://www.qiagen.com/it/shop/genes-and-pathways/data-analysis-center) The expression profile of the glioblastoma was compared with of each DSRCT. After appropriate corrections for preamplified samples, all of the analyses passed the quality control criteria. The data were normalised by using a housekeeping pool (beta 2 microglobulin, actin B and hypoxanthine-guanine phosphoribosyltranferase). The fold-changes were calculated using the 2 ^−ΔΔ Ct^ method with a value of 2 as a fold regulation cut-off value. The fold-changes are expressed as Log10.

## SUPPLEMENTARY MATERIALS FIGURES AND TABLES















## Abbreviations

DSRCTs: desmoplastic small round cell tumors
GEP: gene expression profiling
MErT: emsenchymal epithelial reverse transition
EMT: epithelial mesenchymal transition
AR: androgen receptor
Tf: transcription factor
CSCs: cancer stem cells
CAFs: cancer associated fibroblasts
MSCs: mesenchymal stem cells
ISH: *in situ* hybridisation
IHC: immunohistochemistry
GSEA: gene set enrichment analysis
