# Supplementary material for: New transcriptional-based insights into the pathogenesis of desmoplastic small round cell tumors (DSRCTs)
Source: Oncotarget. 2017 Mar 22;8(20):32492–504. doi: 10.18632/oncotarget.16477 (PMC5464804; doi:10.18632/oncotarget.16477)
Supplement: Supplementary file 1 [file oncotarget-08-32492-s001.pdf]

New transcriptional-based insights into the pathogenesis of desmoplastic small round cell tumors (DSRCTs)

Supplementary Material

SUPPLEMENTARY METHODS

IHC: antibodies and conditions.

P-cadherin (#2130s, Cell Signaling Technology, 1:50, extended CC1 buffer, optiview DAB); E-cadherin (M3612, Dako, 1:50, extended CC1 buffer, optiview DAB); SLUG (#9585s, Cell Signaling Technology, 1:50, extended CC1 buffer, optiview DAB); ZEB-1 (HPA027524, Sigma, 1:200, standard CC1 buffer, ultraview DAB); CD163 (NCL-CD163, Leica, 1:100, standard CC1 buffer, ultraview DAB); PD-L1 (SP-140, Roche, 1:50, CC1 buffer, optiview DAB) were performed using Ventana Benchmark ULTRA platform.

Desmin (IR606, Dako, 1:400, high 15' EDTA 15' linker mouse); CD3 (IS503, Dako, 1:400, high 15' EDTA 15' linker rabbit); CD20 (IR604, Dako, 1:400, high 15' EDTA 15' linker mouse); AR (M3562, Dako, 1:25, high 15' EDTA 15' linker mouse); β-catenin (610154, BD transduction, 1:1000, high 30' EDTA 15' linker mouse); PDGFRA (sc-338, Santa Cruz Biotechnology, 1:200, high 15' EDTA 15' linker rabbit), were performed using Dako Autostainer Link 48.

CD14 (MS-1080, Thermofisher, 1:10) was performed using a pressure cooker heating Dako, 110°C, 20'EDTA buffer, 4°C overnight incubation.

miRNA *in situ* hybridisation (ISH) evaluation

The positive staining signals for miRNAs were identified as brown dots usually localised in the cytoplasm. The size and intensity of the dots varied from case to case, and also depended on the miRNA. miRNA expression in tumor cells was evaluated using a 1-16 scoring system in which the percentage of positive cells (1 = <25%; 2 = 25-50%; 3 = 50-75%; 4 = 75-100%) was multiplied by the number of signals per cell (1 = 1-3 dots; 2 = 4-6 dots; 3 = 6-9 dots;4 = >10 dots).

ISLR/Meflin mRNA ISH

For each case a negative control probe (bacterial gene 4-hydroxy-tetrahydrodipicolinate reductase [DapB] #310043, Advanced Cell Diagnostics) and a positive control probe for evidence of preserved RNA (housekeeping gene ubiquitin C [UbC] #310041, Advanced Cell Diagnostics) was included. ISH was performed manually using the RNAscope 2.5 High Definition detection kit 12 (brown) (Advanced Cell Diagnostics, Inc., Hayward, CA) in accordance with the manufacturer's instructions. Briefly, 5 µm FFPE tissue sections were incubated with hydrogen peroxide for 10 minutes at room temperature (RT). The slides then were boiled in Target Retrieval 1X buffer for 45 minutes, followed by incubation with Protease Plus for 30 minutes at 40°C, and then hybridised with the relevant probes for two hours at 40°C, followed by successive incubations with Amp1-Amp6 reagents, colour development with 3,3'-diaminobenzine (DAB), and counterstaining with hemotoxylin. The positive control was a gastrointestinal stromal tumor (GIST) with a high level of *PDGFRA* mRNA expression. Positive staining signals were identified as brown, punctuate dots in the cytoplasm and/or nucleus.

ISRL/Meflin mRNA ISH scoring

ISRL/Meflin mRNA was analysed by using a scoring system that categorised the cases into five grades depending on the number of dots visualised under a bright-field microscope: 0 = no staining or <1 dot every 10 cells; 1+: 1-3 dots/cell; 2+: 4-10 dots/cell with very few dot clusters; 3+: >10 dots/cell with <10% of positive cells having dot clusters; and 4+: >10 dots/cell with >10% of positive cells having dot clusters.

For Supplementary Tables 1-6 see in Supplementary Files.

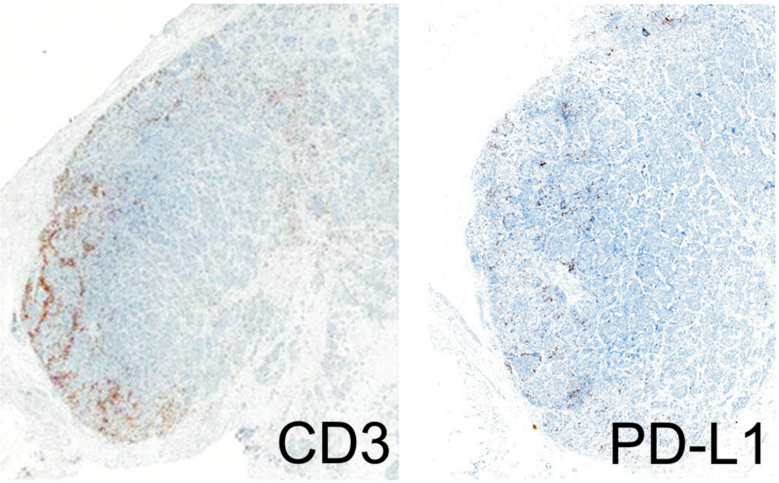

Supplementary Figure 1: Small CD3-positive and a less extensive PD-L1 immunolabelled area from the surgical sample of DSRCT 2 case.

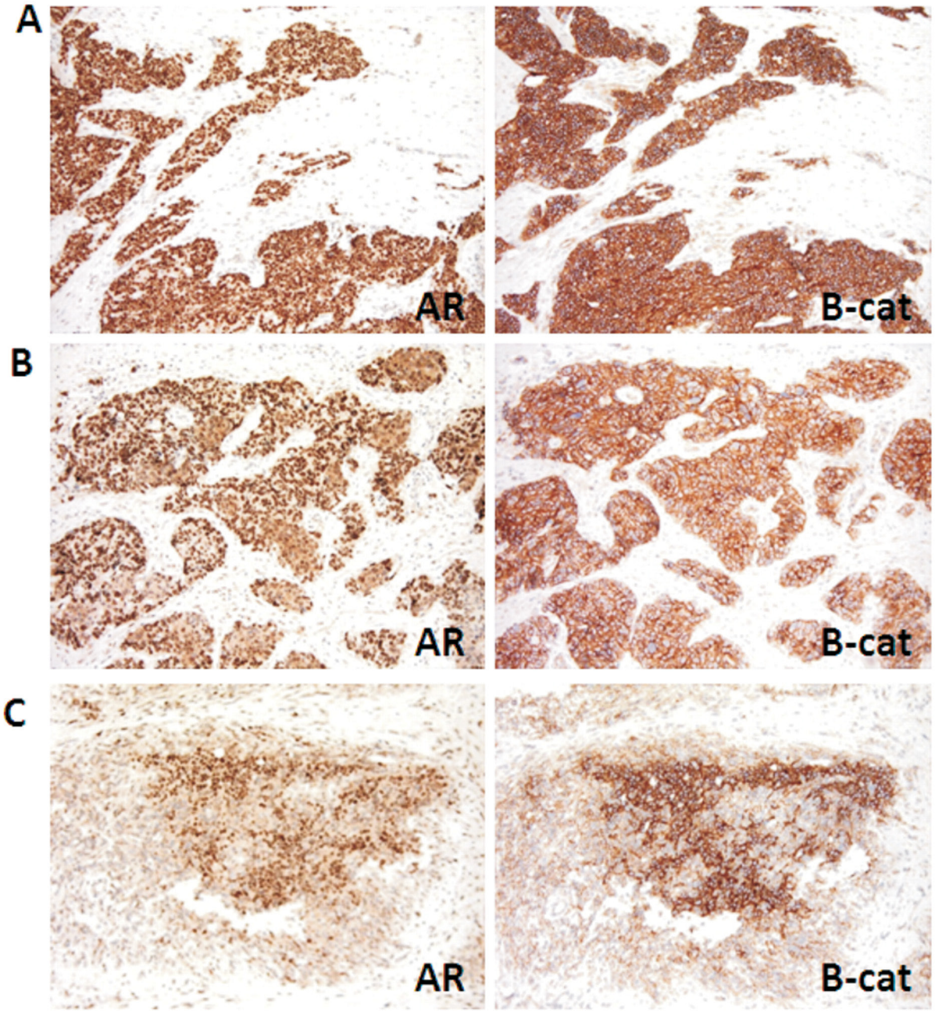

Supplementary Figure 2: AR and β-catenin IHC in DSRCT 4 (A), DSRCT 2 (B) and DSRCT 6 (C) cases. Nuclear AR and membrane β-catenin immunostaining on consecutive sections revealed two superimposable patterns of decoration in the two cases belonging to group 1 (A and B) and one belonging to group 2 (C).
