# Supplementary material for: New transcriptional-based insights into the pathogenesis of desmoplastic small round cell tumors (DSRCTs)
Source: Oncotarget. 2017 Mar 22;8(20):32492–504. doi: 10.18632/oncotarget.16477 (PMC5464804; doi:10.18632/oncotarget.16477)
Supplement: Supplementary file 2 [file oncotarget-08-32492-s002.doc]

| **Supplementary table 1. Differentially expressed genes by class comparison** | | | | | | |
| --- | --- | --- | --- | --- | --- | --- |
| **UniqueID** | **Accession** | **Symbol** | **EntrezID** | **Fold-change Cl1 vs Cl2** | **Parametric p-value** | **FDR** |
| ILMN_1769129 | [NM_006274](http://www.ncbi.nlm.nih.gov/entrez/query.fcgi?db=Nucleotide&term=NM_006274) | [CCL19](http://www.ncbi.nlm.nih.gov/entrez/query.fcgi?cmd=search&db=gene&term=CCL19) | [6363](http://www.ncbi.nlm.nih.gov/sites/entrez?db=gene&cmd=search&term=6363) | 6 | 0,0001469 | 0,043 |
| ILMN_1727605 | [NM_002031](http://www.ncbi.nlm.nih.gov/entrez/query.fcgi?db=Nucleotide&term=NM_002031) | [FRK](http://www.ncbi.nlm.nih.gov/entrez/query.fcgi?cmd=search&db=gene&term=FRK) | [2444](http://www.ncbi.nlm.nih.gov/sites/entrez?db=gene&cmd=search&term=2444) | 2,92 | 0,0002724 | 0,043 |
| ILMN_2395981 | [NM_152501](http://www.ncbi.nlm.nih.gov/entrez/query.fcgi?db=Nucleotide&term=NM_152501) | [PYHIN1](http://www.ncbi.nlm.nih.gov/entrez/query.fcgi?cmd=search&db=gene&term=PYHIN1) | [149628](http://www.ncbi.nlm.nih.gov/sites/entrez?db=gene&cmd=search&term=149628) | 5,35 | 0,0003966 | 0,043 |
| ILMN_1680339 | [NM_006207](http://www.ncbi.nlm.nih.gov/entrez/query.fcgi?db=Nucleotide&term=NM_006207) | [PDGFRL](http://www.ncbi.nlm.nih.gov/entrez/query.fcgi?cmd=search&db=gene&term=PDGFRL) | [5157](http://www.ncbi.nlm.nih.gov/sites/entrez?db=gene&cmd=search&term=5157) | 3,96 | 0,0003986 | 0,043 |
| ILMN_2261627 | [NM_005214](http://www.ncbi.nlm.nih.gov/entrez/query.fcgi?db=Nucleotide&term=NM_005214) | [CTLA4](http://www.ncbi.nlm.nih.gov/entrez/query.fcgi?cmd=search&db=gene&term=CTLA4) | [1493](http://www.ncbi.nlm.nih.gov/sites/entrez?db=gene&cmd=search&term=1493) | 5,27 | 0,0004188 | 0,043 |
| ILMN_1746085 | [NM_000598](http://www.ncbi.nlm.nih.gov/entrez/query.fcgi?db=Nucleotide&term=NM_000598) | [IGFBP3](http://www.ncbi.nlm.nih.gov/entrez/query.fcgi?cmd=search&db=gene&term=IGFBP3) | [3486](http://www.ncbi.nlm.nih.gov/sites/entrez?db=gene&cmd=search&term=3486) | 2,94 | 0,0004351 | 0,043 |
| ILMN_1815093 | [NM_001005325](http://www.ncbi.nlm.nih.gov/entrez/query.fcgi?db=Nucleotide&term=NM_001005325) | [OR6M1](http://www.ncbi.nlm.nih.gov/entrez/query.fcgi?cmd=search&db=gene&term=OR6M1) | [390261](http://www.ncbi.nlm.nih.gov/sites/entrez?db=gene&cmd=search&term=390261) | 3,96 | 0,0004416 | 0,043 |
| ILMN_1707591 | [NM_024873](http://www.ncbi.nlm.nih.gov/entrez/query.fcgi?db=Nucleotide&term=NM_024873) | [TNIP3](http://www.ncbi.nlm.nih.gov/entrez/query.fcgi?cmd=search&db=gene&term=TNIP3) | [79931](http://www.ncbi.nlm.nih.gov/sites/entrez?db=gene&cmd=search&term=79931) | 5,65 | 0,0006432 | 0,043 |
| ILMN_1774982 | [NM_145057](http://www.ncbi.nlm.nih.gov/entrez/query.fcgi?db=Nucleotide&term=NM_145057) | [CDC42EP5](http://www.ncbi.nlm.nih.gov/entrez/query.fcgi?cmd=search&db=gene&term=CDC42EP5) | [148170](http://www.ncbi.nlm.nih.gov/sites/entrez?db=gene&cmd=search&term=148170) | 3,87 | 0,0007672 | 0,043 |
| ILMN_1740385 | [NM_014956](http://www.ncbi.nlm.nih.gov/entrez/query.fcgi?db=Nucleotide&term=NM_014956) | [CEP164](http://www.ncbi.nlm.nih.gov/entrez/query.fcgi?cmd=search&db=gene&term=CEP164) | [22897](http://www.ncbi.nlm.nih.gov/sites/entrez?db=gene&cmd=search&term=22897) | 0,15 | 0,0008565 | 0,043 |
| ILMN_2366212 | [NM_001039933](http://www.ncbi.nlm.nih.gov/entrez/query.fcgi?db=Nucleotide&term=NM_001039933) | [CD79B](http://www.ncbi.nlm.nih.gov/entrez/query.fcgi?cmd=search&db=gene&term=CD79B) | [974](http://www.ncbi.nlm.nih.gov/sites/entrez?db=gene&cmd=search&term=974) | 5,25 | 0,0008729 | 0,043 |
| ILMN_1761858 | [NM_033290](http://www.ncbi.nlm.nih.gov/entrez/query.fcgi?db=Nucleotide&term=NM_033290) | [MID1](http://www.ncbi.nlm.nih.gov/entrez/query.fcgi?cmd=search&db=gene&term=MID1) | [4281](http://www.ncbi.nlm.nih.gov/sites/entrez?db=gene&cmd=search&term=4281) | 3,55 | 0,000879 | 0,065 |
| ILMN_1779324 | [NM_006144](http://www.ncbi.nlm.nih.gov/entrez/query.fcgi?db=Nucleotide&term=NM_006144) | [GZMA](http://www.ncbi.nlm.nih.gov/entrez/query.fcgi?cmd=search&db=gene&term=GZMA) | [3001](http://www.ncbi.nlm.nih.gov/sites/entrez?db=gene&cmd=search&term=3001) | 5,12 | 0,0009386 | 0,065 |
| ILMN_1802611 | [NM_015001](http://www.ncbi.nlm.nih.gov/entrez/query.fcgi?db=Nucleotide&term=NM_015001) | [SPEN](http://www.ncbi.nlm.nih.gov/entrez/query.fcgi?cmd=search&db=gene&term=SPEN) | [23013](http://www.ncbi.nlm.nih.gov/sites/entrez?db=gene&cmd=search&term=23013) | 0,26 | 0,0011817 | 0,07 |
| ILMN_1733276 | [NM_006639](http://www.ncbi.nlm.nih.gov/entrez/query.fcgi?db=Nucleotide&term=NM_006639) | [CYSLTR1](http://www.ncbi.nlm.nih.gov/entrez/query.fcgi?cmd=search&db=gene&term=CYSLTR1) | [10800](http://www.ncbi.nlm.nih.gov/sites/entrez?db=gene&cmd=search&term=10800) | 4,21 | 0,0013226 | 0,07 |
| ILMN_2044081 | [NM_001040704](http://www.ncbi.nlm.nih.gov/entrez/query.fcgi?db=Nucleotide&term=NM_001040704) | [DEFB106B](http://www.ncbi.nlm.nih.gov/entrez/query.fcgi?cmd=search&db=gene&term=DEFB106B) | [503841](http://www.ncbi.nlm.nih.gov/sites/entrez?db=gene&cmd=search&term=503841) | 3,23 | 0,0013304 | 0,07 |
| ILMN_1792538 | [NM_006137](http://www.ncbi.nlm.nih.gov/entrez/query.fcgi?db=Nucleotide&term=NM_006137) | [CD7](http://www.ncbi.nlm.nih.gov/entrez/query.fcgi?cmd=search&db=gene&term=CD7) | [924](http://www.ncbi.nlm.nih.gov/sites/entrez?db=gene&cmd=search&term=924) | 6,43 | 0,0014039 | 0,07 |
| ILMN_1786278 | [NM_015398](http://www.ncbi.nlm.nih.gov/entrez/query.fcgi?db=Nucleotide&term=NM_015398) | [FAM149A](http://www.ncbi.nlm.nih.gov/entrez/query.fcgi?cmd=search&db=gene&term=FAM149A) | [25854](http://www.ncbi.nlm.nih.gov/sites/entrez?db=gene&cmd=search&term=25854) | 2,72 | 0,0014324 | 0,07 |
| ILMN_2342579 | [NM_002185](http://www.ncbi.nlm.nih.gov/entrez/query.fcgi?db=Nucleotide&term=NM_002185) | [IL7R](http://www.ncbi.nlm.nih.gov/entrez/query.fcgi?cmd=search&db=gene&term=IL7R) | [3575](http://www.ncbi.nlm.nih.gov/sites/entrez?db=gene&cmd=search&term=3575) | 2,79 | 0,001466 | 0,07 |
| ILMN_1720440 | [NM_133636](http://www.ncbi.nlm.nih.gov/entrez/query.fcgi?db=Nucleotide&term=NM_133636) | [HELQ](http://www.ncbi.nlm.nih.gov/entrez/query.fcgi?cmd=search&db=gene&term=HELQ) | [113510](http://www.ncbi.nlm.nih.gov/sites/entrez?db=gene&cmd=search&term=113510) | 0,35 | 0,0015722 | 0,07 |
| ILMN_2088124 | [NM_152680](http://www.ncbi.nlm.nih.gov/entrez/query.fcgi?db=Nucleotide&term=NM_152680) | [TMEM154](http://www.ncbi.nlm.nih.gov/entrez/query.fcgi?cmd=search&db=gene&term=TMEM154) | [201799](http://www.ncbi.nlm.nih.gov/sites/entrez?db=gene&cmd=search&term=201799) | 3,64 | 0,0016151 | 0,07 |
| ILMN_1683757 | [NM_020982](http://www.ncbi.nlm.nih.gov/entrez/query.fcgi?db=Nucleotide&term=NM_020982) | [CLDN9](http://www.ncbi.nlm.nih.gov/entrez/query.fcgi?cmd=search&db=gene&term=CLDN9) | [9080](http://www.ncbi.nlm.nih.gov/sites/entrez?db=gene&cmd=search&term=9080) | 5,52 | 0,0018897 | 0,07 |
| ILMN_1784774 | [NM_014499](http://www.ncbi.nlm.nih.gov/entrez/query.fcgi?db=Nucleotide&term=NM_014499) | [P2RY10](http://www.ncbi.nlm.nih.gov/entrez/query.fcgi?cmd=search&db=gene&term=P2RY10) | [27334](http://www.ncbi.nlm.nih.gov/sites/entrez?db=gene&cmd=search&term=27334) | 4,13 | 0,0019972 | 0,07 |
| ILMN_3237069 | [NM_001100817](http://www.ncbi.nlm.nih.gov/entrez/query.fcgi?db=Nucleotide&term=NM_001100817) | [TCEB3CL](http://www.ncbi.nlm.nih.gov/entrez/query.fcgi?cmd=search&db=gene&term=TCEB3CL) | [728929](http://www.ncbi.nlm.nih.gov/sites/entrez?db=gene&cmd=search&term=728929) | 5,86 | 0,0021081 | 0,07 |
| ILMN_2231928 | [NM_002463](http://www.ncbi.nlm.nih.gov/entrez/query.fcgi?db=Nucleotide&term=NM_002463) | [MX2](http://www.ncbi.nlm.nih.gov/entrez/query.fcgi?cmd=search&db=gene&term=MX2) | [4600](http://www.ncbi.nlm.nih.gov/sites/entrez?db=gene&cmd=search&term=4600) | 4,87 | 0,0021538 | 0,07 |
| ILMN_2349658 | [NM_007311](http://www.ncbi.nlm.nih.gov/entrez/query.fcgi?db=Nucleotide&term=NM_007311) | [TSPO](http://www.ncbi.nlm.nih.gov/entrez/query.fcgi?cmd=search&db=gene&term=TSPO) | [706](http://www.ncbi.nlm.nih.gov/sites/entrez?db=gene&cmd=search&term=706) | 2,78 | 0,0021881 | 0,07 |
| ILMN_2125747 | [NR_002454](http://www.ncbi.nlm.nih.gov/entrez/query.fcgi?db=Nucleotide&term=NR_002454) | [LOC606724](http://www.ncbi.nlm.nih.gov/entrez/query.fcgi?cmd=search&db=gene&term=LOC606724) | [606724](http://www.ncbi.nlm.nih.gov/sites/entrez?db=gene&cmd=search&term=606724) | 4,53 | 0,0022093 | 0,07 |
| ILMN_1675191 | [NM_152687](http://www.ncbi.nlm.nih.gov/entrez/query.fcgi?db=Nucleotide&term=NM_152687) | [GAPT](http://www.ncbi.nlm.nih.gov/entrez/query.fcgi?cmd=search&db=gene&term=GAPT) | [202309](http://www.ncbi.nlm.nih.gov/sites/entrez?db=gene&cmd=search&term=202309) | 3,55 | 0,0022844 | 0,07 |
| ILMN_1727532 | [NM_020190](http://www.ncbi.nlm.nih.gov/entrez/query.fcgi?db=Nucleotide&term=NM_020190) | [OLFML3](http://www.ncbi.nlm.nih.gov/entrez/query.fcgi?cmd=search&db=gene&term=OLFML3) | [56944](http://www.ncbi.nlm.nih.gov/sites/entrez?db=gene&cmd=search&term=56944) | 3,06 | 0,0023046 | 0,07 |
| ILMN_2111229 | [NM_004758](http://www.ncbi.nlm.nih.gov/entrez/query.fcgi?db=Nucleotide&term=NM_004758) | [BZRAP1](http://www.ncbi.nlm.nih.gov/entrez/query.fcgi?cmd=search&db=gene&term=BZRAP1) | [9256](http://www.ncbi.nlm.nih.gov/sites/entrez?db=gene&cmd=search&term=9256) | 0,31 | 0,002321 | 0,07 |
| ILMN_1760208 | [NM_024034](http://www.ncbi.nlm.nih.gov/entrez/query.fcgi?db=Nucleotide&term=NM_024034) | [GDAP1L1](http://www.ncbi.nlm.nih.gov/entrez/query.fcgi?cmd=search&db=gene&term=GDAP1L1) | [78997](http://www.ncbi.nlm.nih.gov/sites/entrez?db=gene&cmd=search&term=78997) | 3,7 | 0,0023766 | 0,07 |
| ILMN_2068257 | [NM_032348](http://www.ncbi.nlm.nih.gov/entrez/query.fcgi?db=Nucleotide&term=NM_032348) | [MXRA8](http://www.ncbi.nlm.nih.gov/entrez/query.fcgi?cmd=search&db=gene&term=MXRA8) | [54587](http://www.ncbi.nlm.nih.gov/sites/entrez?db=gene&cmd=search&term=54587) | 3,63 | 0,0025907 | 0,07 |
| ILMN_1651692 | [NM_005990](http://www.ncbi.nlm.nih.gov/entrez/query.fcgi?db=Nucleotide&term=NM_005990) | [STK10](http://www.ncbi.nlm.nih.gov/entrez/query.fcgi?cmd=search&db=gene&term=STK10) | [6793](http://www.ncbi.nlm.nih.gov/sites/entrez?db=gene&cmd=search&term=6793) | 3,07 | 0,0026514 | 0,11 |
| ILMN_1763442 | [NM_020717](http://www.ncbi.nlm.nih.gov/entrez/query.fcgi?db=Nucleotide&term=NM_020717) | [KIAA1202](http://www.ncbi.nlm.nih.gov/entrez/query.fcgi?cmd=search&db=gene&term=KIAA1202) | [57477](http://www.ncbi.nlm.nih.gov/sites/entrez?db=gene&cmd=search&term=57477) | 0,37 | 0,0026632 | 0,11 |
| ILMN_1723962 | [NM_020169](http://www.ncbi.nlm.nih.gov/entrez/query.fcgi?db=Nucleotide&term=NM_020169) | [LXN](http://www.ncbi.nlm.nih.gov/entrez/query.fcgi?cmd=search&db=gene&term=LXN) | [56925](http://www.ncbi.nlm.nih.gov/sites/entrez?db=gene&cmd=search&term=56925) | 3,03 | 0,0026688 | 0,11 |
| ILMN_1777949 | [NM_173080](http://www.ncbi.nlm.nih.gov/entrez/query.fcgi?db=Nucleotide&term=NM_173080) | [SPRR4](http://www.ncbi.nlm.nih.gov/entrez/query.fcgi?cmd=search&db=gene&term=SPRR4) | [163778](http://www.ncbi.nlm.nih.gov/sites/entrez?db=gene&cmd=search&term=163778) | 5,7 | 0,0026985 | 0,11 |
| ILMN_1805098 | [NM_000924](http://www.ncbi.nlm.nih.gov/entrez/query.fcgi?db=Nucleotide&term=NM_000924) | [PDE1B](http://www.ncbi.nlm.nih.gov/entrez/query.fcgi?cmd=search&db=gene&term=PDE1B) | [5153](http://www.ncbi.nlm.nih.gov/sites/entrez?db=gene&cmd=search&term=5153) | 3,13 | 0,0027508 | 0,11 |
| ILMN_1773352 | [NM_002985](http://www.ncbi.nlm.nih.gov/entrez/query.fcgi?db=Nucleotide&term=NM_002985) | [CCL5](http://www.ncbi.nlm.nih.gov/entrez/query.fcgi?cmd=search&db=gene&term=CCL5) | [6352](http://www.ncbi.nlm.nih.gov/sites/entrez?db=gene&cmd=search&term=6352) | 7,02 | 0,0028832 | 0,11 |
| ILMN_1781951 | [NM_014191](http://www.ncbi.nlm.nih.gov/entrez/query.fcgi?db=Nucleotide&term=NM_014191) | [SCN8A](http://www.ncbi.nlm.nih.gov/entrez/query.fcgi?cmd=search&db=gene&term=SCN8A) | [6334](http://www.ncbi.nlm.nih.gov/sites/entrez?db=gene&cmd=search&term=6334) | 0,36 | 0,0028929 | 0,11 |
| ILMN_1712628 | [NM_016328](http://www.ncbi.nlm.nih.gov/entrez/query.fcgi?db=Nucleotide&term=NM_016328) | [GTF2IRD1](http://www.ncbi.nlm.nih.gov/entrez/query.fcgi?cmd=search&db=gene&term=GTF2IRD1) | [9569](http://www.ncbi.nlm.nih.gov/sites/entrez?db=gene&cmd=search&term=9569) | 0,34 | 0,0029183 | 0,11 |
| ILMN_1668910 | [NM_014430](http://www.ncbi.nlm.nih.gov/entrez/query.fcgi?db=Nucleotide&term=NM_014430) | [CIDEB](http://www.ncbi.nlm.nih.gov/entrez/query.fcgi?cmd=search&db=gene&term=CIDEB) | [27141](http://www.ncbi.nlm.nih.gov/sites/entrez?db=gene&cmd=search&term=27141) | 2,78 | 0,0029764 | 0,11 |
| ILMN_1804901 | [NM_173170](http://www.ncbi.nlm.nih.gov/entrez/query.fcgi?db=Nucleotide&term=NM_173170) | [IL1F5](http://www.ncbi.nlm.nih.gov/entrez/query.fcgi?cmd=search&db=gene&term=IL1F5) | [26525](http://www.ncbi.nlm.nih.gov/sites/entrez?db=gene&cmd=search&term=26525) | 6,12 | 0,0031604 | 0,11 |
| ILMN_1663683 | [NM_014799](http://www.ncbi.nlm.nih.gov/entrez/query.fcgi?db=Nucleotide&term=NM_014799) | [HEPH](http://www.ncbi.nlm.nih.gov/entrez/query.fcgi?cmd=search&db=gene&term=HEPH) | [9843](http://www.ncbi.nlm.nih.gov/sites/entrez?db=gene&cmd=search&term=9843) | 4,04 | 0,0031826 | 0,11 |
| ILMN_2354381 | [NM_000305](http://www.ncbi.nlm.nih.gov/entrez/query.fcgi?db=Nucleotide&term=NM_000305) | [PON2](http://www.ncbi.nlm.nih.gov/entrez/query.fcgi?cmd=search&db=gene&term=PON2) | [5445](http://www.ncbi.nlm.nih.gov/sites/entrez?db=gene&cmd=search&term=5445) | 0,24 | 0,0033782 | 0,11 |
| ILMN_1738725 | [NM_002309](http://www.ncbi.nlm.nih.gov/entrez/query.fcgi?db=Nucleotide&term=NM_002309) | [LIF](http://www.ncbi.nlm.nih.gov/entrez/query.fcgi?cmd=search&db=gene&term=LIF) | [3976](http://www.ncbi.nlm.nih.gov/sites/entrez?db=gene&cmd=search&term=3976) | 3,54 | 0,003396 | 0,11 |
| ILMN_2368530 | [NM_001012633](http://www.ncbi.nlm.nih.gov/entrez/query.fcgi?db=Nucleotide&term=NM_001012633) | [IL32](http://www.ncbi.nlm.nih.gov/entrez/query.fcgi?cmd=search&db=gene&term=IL32) | [9235](http://www.ncbi.nlm.nih.gov/sites/entrez?db=gene&cmd=search&term=9235) | 6,3 | 0,0033997 | 0,11 |
| ILMN_1678517 | [NM_016234](http://www.ncbi.nlm.nih.gov/entrez/query.fcgi?db=Nucleotide&term=NM_016234) | [ACSL5](http://www.ncbi.nlm.nih.gov/entrez/query.fcgi?cmd=search&db=gene&term=ACSL5) | [51703](http://www.ncbi.nlm.nih.gov/sites/entrez?db=gene&cmd=search&term=51703) | 3,96 | 0,003456 | 0,11 |
| ILMN_1739397 | [NM_053274](http://www.ncbi.nlm.nih.gov/entrez/query.fcgi?db=Nucleotide&term=NM_053274) | [GLMN](http://www.ncbi.nlm.nih.gov/entrez/query.fcgi?cmd=search&db=gene&term=GLMN) | [11146](http://www.ncbi.nlm.nih.gov/sites/entrez?db=gene&cmd=search&term=11146) | 0,34 | 0,0034638 | 0,11 |
| ILMN_1682332 | [NM_016815](http://www.ncbi.nlm.nih.gov/entrez/query.fcgi?db=Nucleotide&term=NM_016815) | [GYPC](http://www.ncbi.nlm.nih.gov/entrez/query.fcgi?cmd=search&db=gene&term=GYPC) | [2995](http://www.ncbi.nlm.nih.gov/sites/entrez?db=gene&cmd=search&term=2995) | 5,76 | 0,0034808 | 0,11 |
| ILMN_1663866 | [NM_000358](http://www.ncbi.nlm.nih.gov/entrez/query.fcgi?db=Nucleotide&term=NM_000358) | [TGFBI](http://www.ncbi.nlm.nih.gov/entrez/query.fcgi?cmd=search&db=gene&term=TGFBI) | [7045](http://www.ncbi.nlm.nih.gov/sites/entrez?db=gene&cmd=search&term=7045) | 4,19 | 0,0035307 | 0,11 |
| ILMN_3246071 | [NM_001130405](http://www.ncbi.nlm.nih.gov/entrez/query.fcgi?db=Nucleotide&term=NM_001130405) | [PRR20C](http://www.ncbi.nlm.nih.gov/entrez/query.fcgi?cmd=search&db=gene&term=PRR20C) | [729240](http://www.ncbi.nlm.nih.gov/sites/entrez?db=gene&cmd=search&term=729240) | 11,79 | 0,0036474 | 0,11 |
| ILMN_1758728 | [NM_004629](http://www.ncbi.nlm.nih.gov/entrez/query.fcgi?db=Nucleotide&term=NM_004629) | [FANCG](http://www.ncbi.nlm.nih.gov/entrez/query.fcgi?cmd=search&db=gene&term=FANCG) | [2189](http://www.ncbi.nlm.nih.gov/sites/entrez?db=gene&cmd=search&term=2189) | 0,33 | 0,0037044 | 0,11 |
| ILMN_1770433 | [NM_002649](http://www.ncbi.nlm.nih.gov/entrez/query.fcgi?db=Nucleotide&term=NM_002649) | [PIK3CG](http://www.ncbi.nlm.nih.gov/entrez/query.fcgi?cmd=search&db=gene&term=PIK3CG) | [5294](http://www.ncbi.nlm.nih.gov/sites/entrez?db=gene&cmd=search&term=5294) | 2,77 | 0,003717 | 0,11 |
| ILMN_1747593 | [NM_005545](http://www.ncbi.nlm.nih.gov/entrez/query.fcgi?db=Nucleotide&term=NM_005545) | [ISLR](http://www.ncbi.nlm.nih.gov/entrez/query.fcgi?cmd=search&db=gene&term=ISLR) | [3671](http://www.ncbi.nlm.nih.gov/sites/entrez?db=gene&cmd=search&term=3671) | 4,78 | 0,0037228 | 0,11 |
| ILMN_1664543 | [NM_001031683](http://www.ncbi.nlm.nih.gov/entrez/query.fcgi?db=Nucleotide&term=NM_001031683) | [IFIT3](http://www.ncbi.nlm.nih.gov/entrez/query.fcgi?cmd=search&db=gene&term=IFIT3) | [3437](http://www.ncbi.nlm.nih.gov/sites/entrez?db=gene&cmd=search&term=3437) | 2,99 | 0,0037384 | 0,11 |
| ILMN_1779257 | [NM_001250](http://www.ncbi.nlm.nih.gov/entrez/query.fcgi?db=Nucleotide&term=NM_001250) | [CD40](http://www.ncbi.nlm.nih.gov/entrez/query.fcgi?cmd=search&db=gene&term=CD40) | [958](http://www.ncbi.nlm.nih.gov/sites/entrez?db=gene&cmd=search&term=958) | 4,13 | 0,0038034 | 0,11 |
| ILMN_1759175 | [NM_007207](http://www.ncbi.nlm.nih.gov/entrez/query.fcgi?db=Nucleotide&term=NM_007207) | [DUSP10](http://www.ncbi.nlm.nih.gov/entrez/query.fcgi?cmd=search&db=gene&term=DUSP10) | [11221](http://www.ncbi.nlm.nih.gov/sites/entrez?db=gene&cmd=search&term=11221) | 3,11 | 0,0038608 | 0,12 |
| ILMN_1692332 | [NM_001139](http://www.ncbi.nlm.nih.gov/entrez/query.fcgi?db=Nucleotide&term=NM_001139) | [ALOX12B](http://www.ncbi.nlm.nih.gov/entrez/query.fcgi?cmd=search&db=gene&term=ALOX12B) | [242](http://www.ncbi.nlm.nih.gov/sites/entrez?db=gene&cmd=search&term=242) | 5,44 | 0,0038826 | 0,12 |
| ILMN_1667232 | [NM_014511](http://www.ncbi.nlm.nih.gov/entrez/query.fcgi?db=Nucleotide&term=NM_014511) | [KIR2DL3](http://www.ncbi.nlm.nih.gov/entrez/query.fcgi?cmd=search&db=gene&term=KIR2DL3) | [3804](http://www.ncbi.nlm.nih.gov/sites/entrez?db=gene&cmd=search&term=3804) | 4,17 | 0,0039639 | 0,12 |
| ILMN_1778064 | [NM_007076](http://www.ncbi.nlm.nih.gov/entrez/query.fcgi?db=Nucleotide&term=NM_007076) | [FICD](http://www.ncbi.nlm.nih.gov/entrez/query.fcgi?cmd=search&db=gene&term=FICD) | [11153](http://www.ncbi.nlm.nih.gov/sites/entrez?db=gene&cmd=search&term=11153) | 2,93 | 0,0039788 | 0,12 |
| ILMN_1758315 | [NM_173653](http://www.ncbi.nlm.nih.gov/entrez/query.fcgi?db=Nucleotide&term=NM_173653) | [SLC9A9](http://www.ncbi.nlm.nih.gov/entrez/query.fcgi?cmd=search&db=gene&term=SLC9A9) | [285195](http://www.ncbi.nlm.nih.gov/sites/entrez?db=gene&cmd=search&term=285195) | 2,94 | 0,0039906 | 0,12 |
| ILMN_1665647 | [NM_005582](http://www.ncbi.nlm.nih.gov/entrez/query.fcgi?db=Nucleotide&term=NM_005582) | [CD180](http://www.ncbi.nlm.nih.gov/entrez/query.fcgi?cmd=search&db=gene&term=CD180) | [4064](http://www.ncbi.nlm.nih.gov/sites/entrez?db=gene&cmd=search&term=4064) | 3,14 | 0,0040801 | 0,12 |
| ILMN_1781721 | [NM_022779](http://www.ncbi.nlm.nih.gov/entrez/query.fcgi?db=Nucleotide&term=NM_022779) | [DDX31](http://www.ncbi.nlm.nih.gov/entrez/query.fcgi?cmd=search&db=gene&term=DDX31) | [64794](http://www.ncbi.nlm.nih.gov/sites/entrez?db=gene&cmd=search&term=64794) | 0,3 | 0,0041651 | 0,12 |
| ILMN_2083588 | [NM_001008237](http://www.ncbi.nlm.nih.gov/entrez/query.fcgi?db=Nucleotide&term=NM_001008237) | [TTC32](http://www.ncbi.nlm.nih.gov/entrez/query.fcgi?cmd=search&db=gene&term=TTC32) | [130502](http://www.ncbi.nlm.nih.gov/sites/entrez?db=gene&cmd=search&term=130502) | 0,32 | 0,0042039 | 0,12 |
| ILMN_3245098 | [NR_002838](http://www.ncbi.nlm.nih.gov/entrez/query.fcgi?db=Nucleotide&term=NR_002838) | [KC6](http://www.ncbi.nlm.nih.gov/entrez/query.fcgi?cmd=search&db=gene&term=KC6) | [641516](http://www.ncbi.nlm.nih.gov/sites/entrez?db=gene&cmd=search&term=641516) | 4,53 | 0,0042277 | 0,12 |
| ILMN_1691743 | [NM_152219](http://www.ncbi.nlm.nih.gov/entrez/query.fcgi?db=Nucleotide&term=NM_152219) | [GJD3](http://www.ncbi.nlm.nih.gov/entrez/query.fcgi?cmd=search&db=gene&term=GJD3) | [125111](http://www.ncbi.nlm.nih.gov/sites/entrez?db=gene&cmd=search&term=125111) | 2,76 | 0,0043072 | 0,12 |
| ILMN_1762115 | [NM_145858](http://www.ncbi.nlm.nih.gov/entrez/query.fcgi?db=Nucleotide&term=NM_145858) | [CRYZL1](http://www.ncbi.nlm.nih.gov/entrez/query.fcgi?cmd=search&db=gene&term=CRYZL1) | [9946](http://www.ncbi.nlm.nih.gov/sites/entrez?db=gene&cmd=search&term=9946) | 0,29 | 0,0043225 | 0,12 |
| ILMN_1763311 | [NM_030572](http://www.ncbi.nlm.nih.gov/entrez/query.fcgi?db=Nucleotide&term=NM_030572) | [C12orf39](http://www.ncbi.nlm.nih.gov/entrez/query.fcgi?cmd=search&db=gene&term=C12orf39) | [80763](http://www.ncbi.nlm.nih.gov/sites/entrez?db=gene&cmd=search&term=80763) | 0,3 | 0,0043272 | 0,12 |
| ILMN_1732023 | [NM_001098173](http://www.ncbi.nlm.nih.gov/entrez/query.fcgi?db=Nucleotide&term=NM_001098173) | [PRDM7](http://www.ncbi.nlm.nih.gov/entrez/query.fcgi?cmd=search&db=gene&term=PRDM7) | [11105](http://www.ncbi.nlm.nih.gov/sites/entrez?db=gene&cmd=search&term=11105) | 3,29 | 0,0043686 | 0,12 |
| ILMN_1713749 | [NM_007074](http://www.ncbi.nlm.nih.gov/entrez/query.fcgi?db=Nucleotide&term=NM_007074) | [CORO1A](http://www.ncbi.nlm.nih.gov/entrez/query.fcgi?cmd=search&db=gene&term=CORO1A) | [11151](http://www.ncbi.nlm.nih.gov/sites/entrez?db=gene&cmd=search&term=11151) | 6,13 | 0,0044492 | 0,12 |
| ILMN_1766169 | [NM_005504](http://www.ncbi.nlm.nih.gov/entrez/query.fcgi?db=Nucleotide&term=NM_005504) | [BCAT1](http://www.ncbi.nlm.nih.gov/entrez/query.fcgi?cmd=search&db=gene&term=BCAT1) | [586](http://www.ncbi.nlm.nih.gov/sites/entrez?db=gene&cmd=search&term=586) | 4,62 | 0,004458 | 0,12 |
| ILMN_1713347 | [NM_006614](http://www.ncbi.nlm.nih.gov/entrez/query.fcgi?db=Nucleotide&term=NM_006614) | [CHL1](http://www.ncbi.nlm.nih.gov/entrez/query.fcgi?cmd=search&db=gene&term=CHL1) | [10752](http://www.ncbi.nlm.nih.gov/sites/entrez?db=gene&cmd=search&term=10752) | 3,21 | 0,0044958 | 0,12 |
| ILMN_1703708 | [NM_001012710](http://www.ncbi.nlm.nih.gov/entrez/query.fcgi?db=Nucleotide&term=NM_001012710) | [KRTAP5-10](http://www.ncbi.nlm.nih.gov/entrez/query.fcgi?cmd=search&db=gene&term=KRTAP5-10) | [387273](http://www.ncbi.nlm.nih.gov/sites/entrez?db=gene&cmd=search&term=387273) | 2,87 | 0,0045719 | 0,12 |
| ILMN_1712506 | [NM_130797](http://www.ncbi.nlm.nih.gov/entrez/query.fcgi?db=Nucleotide&term=NM_130797) | [DPP6](http://www.ncbi.nlm.nih.gov/entrez/query.fcgi?cmd=search&db=gene&term=DPP6) | [1804](http://www.ncbi.nlm.nih.gov/sites/entrez?db=gene&cmd=search&term=1804) | 0,26 | 0,0045758 | 0,12 |
| ILMN_1753789 | [NM_022093](http://www.ncbi.nlm.nih.gov/entrez/query.fcgi?db=Nucleotide&term=NM_022093) | [TNN](http://www.ncbi.nlm.nih.gov/entrez/query.fcgi?cmd=search&db=gene&term=TNN) | [63923](http://www.ncbi.nlm.nih.gov/sites/entrez?db=gene&cmd=search&term=63923) | 5,81 | 0,004715 | 0,12 |
| ILMN_1716563 | [NM_212535](http://www.ncbi.nlm.nih.gov/entrez/query.fcgi?db=Nucleotide&term=NM_212535) | [PRKCB1](http://www.ncbi.nlm.nih.gov/entrez/query.fcgi?cmd=search&db=gene&term=PRKCB1) | [5579](http://www.ncbi.nlm.nih.gov/sites/entrez?db=gene&cmd=search&term=5579) | 3,1 | 0,0049304 | 0,12 |
| ILMN_2138622 | [NM_207495](http://www.ncbi.nlm.nih.gov/entrez/query.fcgi?db=Nucleotide&term=NM_207495) | [DKFZp686I15217](http://www.ncbi.nlm.nih.gov/entrez/query.fcgi?cmd=search&db=gene&term=DKFZp686I15217) | [401232](http://www.ncbi.nlm.nih.gov/sites/entrez?db=gene&cmd=search&term=401232) | 2,82 | 0,0049442 | 0,12 |
