# Supplementary material for: New transcriptional-based insights into the pathogenesis of desmoplastic small round cell tumors (DSRCTs)
Source: Oncotarget. 2017 Mar 22;8(20):32492–504. doi: 10.18632/oncotarget.16477 (PMC5464804; doi:10.18632/oncotarget.16477)
Supplement: Supplementary file 3 [file oncotarget-08-32492-s003.doc]

| **Supplementary table 2. IHC assessment of the immune system biomarkers** | | | | |
| --- | --- | --- | --- | --- |
| **Patient ID** | **CD3** | **CD20** | **CD14** | **CD163** |
| DSRCT 1 | - | - | - | - |
| DSRCT 2 | + | - | + | + |
| DSRCT 3 | - | - | - | - |
| DSRCT 4 | - | - | + | + |
| DSRCT 5 | - | - | - | - |
| DSRCT 6 | - | - | + | + |
| DSRCT 7 | - | - | - | - |
