# Supplementary material for: New transcriptional-based insights into the pathogenesis of desmoplastic small round cell tumors (DSRCTs)
Source: Oncotarget. 2017 Mar 22;8(20):32492–504. doi: 10.18632/oncotarget.16477 (PMC5464804; doi:10.18632/oncotarget.16477)
Supplement: Supplementary file 5 [file oncotarget-08-32492-s005.doc]

| **Supplementary table 4. AR expression/modulation by chimera-module subdivision. _a_** | | | | | | | | | | | |
| --- | --- | --- | --- | --- | --- | --- | --- | --- | --- | --- | --- |
| **patient ID** |  | **miR-200** | **E-cadherin** | **ZEB-1** | **miR-34** | **SLUG** | **Desmin** | **P-cadherin** | **AR** | **B-catenin** | **GEP clusters** |
| DSRCT 4 | **Group 1 (MErT)** | high | +++ | - | high | + | foc | + | +++ | +++ | 2 |
| DSRCT 2 |  | high | ++ | - | high | + | ++het | ++ → - | ++ | ++ | 1 |
|  | | | | | | | | | | | |
| DSRCT 5 | **Group 2 (hybrid/partial)** | int | ++ het | ++ et | int | + | +++ | foc | ++ | ++het | 2 |
| DSRCT 6 |  | int | ++ het | ++ et | int | + | ++het | ++ | + | ++het | 2 |
| DSRCT 1 |  | int/low | + het | + et | int/low | + | ++het | ++ → - | - | ++ | 1 |
|  | | | | | | | | | | | |
| DSRCT 3 | **Group 3 (EMT)** | low | - | ++ | low | + | +++ | +dot | - | + faint | 1 |
| DSRCT 7 |  | low | - | +++ | low | - | ++het | - | - | +het | 2 |
|  |  |  |  |  |  |  |  |  |  |  |  |
| a) the last column indicates GEP cluster subdivision | | | | | | | | | | | |
| int: intermediate; het: heterogeneous; foc: focal | | | | |  |  |  |  |  |  |  |
