# Supplementary material for: New transcriptional-based insights into the pathogenesis of desmoplastic small round cell tumors (DSRCTs)
Source: Oncotarget. 2017 Mar 22;8(20):32492–504. doi: 10.18632/oncotarget.16477 (PMC5464804; doi:10.18632/oncotarget.16477)
Supplement: Supplementary file 6 [file oncotarget-08-32492-s006.doc]

| **Supplementary table 5. DSRCT clinical data** | | | | | |
| --- | --- | --- | --- | --- | --- |
| **Patient ID** | **gender/age (at disease onset)** | **site of disease** | **CT regiment/courses(weeks)** | **administration stop before surgery (weeks)** | **FU (months)** |
| DSRCT 1 | ♂ /17 | DAPD | epirubicin/ifosfamide(6) | 4 | lost |
| DSRCT 2 | ♂ / 32 | DAPD | epirubicin/ifosfamide/ etoposide/CDDP (3) | 3 | AWD (48) lost |
| DSRCT 3 | ♂ / 32 | DAPD | epirubicin/ifosfamide/ etoposide/CDDP (4) | 3 | DOD (36) |
| DSRCT 4 | ♂ / 32 | DAPD | epirubicin/ifosfamide/ etoposide/vincristine (3) | 4 | DOD (11) |
| DSRCT 5 | ♂ / 19 | DAPD | epirubicin/ifosfamide/ etoposide/CDDP (3) | 4 | AWD (8) lost |
| DSRCT 6 | ♂ / 28 | DAPD, M1_a_ | epirubicin/ifosfamide/ etoposide/CDDP (6) | 4 | AWD (20) lost |
| DSRCT 7 | ♂ / 24 | DAPD | epirubicin/ifosfamide/ etoposide/vincristine (4) | 3 | DOD (60) |
| a) M1: synchronous lung metastasis | | |  |  |  |
| DAPD: diffuse abdominopelvic disease; AWD: alive with disease; DOD: dead of disease | | | | |  |
