# Supplementary material for: New transcriptional-based insights into the pathogenesis of desmoplastic small round cell tumors (DSRCTs)
Source: Oncotarget. 2017 Mar 22;8(20):32492–504. doi: 10.18632/oncotarget.16477 (PMC5464804; doi:10.18632/oncotarget.16477)
Supplement: Supplementary file 7 [file oncotarget-08-32492-s007.doc]

| **Supplementary table 6. Human cancer stem cell RT^2^ profiler PCR array raw data** | | | | | | |
| --- | --- | --- | --- | --- | --- | --- |
|  | | **Glioblastoma** | **DSRCT 5** | **DSRCT 1** | **DSRCT 4** | **DSRCT 7** |
| **ABCB5** | A1 | 31,93 | 32,31 | 32,54 | 25,10 | 31,34 |
| **ABCG2** | A2 | 24,96 | 27,44 | 27,64 | 23,68 | 27,57 |
| **ALCAM** | A3 | 24,70 | 25,62 | 26,34 | 21,04 | 28,20 |
| **ALDH1A1** | A4 | 29,07 | 27,90 | 27,49 | 24,35 | 30,97 |
| **ATM** | A5 | 25,33 | 25,70 | 26,56 | 23,75 | 26,72 |
| **ATXN1** | A6 | 25,14 | 26,46 | 27,54 | 24,23 | 30,03 |
| **AXL** | A7 | 24,75 | 22,88 | 22,38 | 21,23 | 22,62 |
| **BM1** | A8 | 28,17 | 28,64 | 30,91 | 23,83 | 29,48 |
| **BMP7** | A9 | 26,76 | 24,91 | 25,90 | 21,95 | 27,64 |
| **CD24** | A10 | 31,57 | 27,73 | 25,60 | 24,32 | 28,45 |
| **CD34** | A11 | 24,00 | 26,90 | 26,47 | 24,04 | 26,44 |
| **CD38** | A12 | Undetermined | 31,19 | 30,64 | 28,22 | Undetermined |
| **CD44** | B1 | 26,28 | 28,91 | 29,41 | 24,83 | 31,78 |
| **CHEK1** | B2 | 25,19 | 25,81 | 27,69 | 25,95 | 27,83 |
| **DACH1** | B3 | 32,09 | Undetermined | 31,96 | 27,14 | 33,21 |
| **DDR1** | B4 | 28,12 | 30,81 | 30,90 | 25,38 | 38,20 |
| **DKK1** | B5 | 32,52 | 32,06 | 31,60 | 28,28 | 32,06 |
| **DLL1** | B6 | 31,72 | Undetermined | Undetermined | 24,11 | Undetermined |
| **DLL4** | B7 | 32,64 | 30,68 | 30,85 | 25,14 | 33,04 |
| **DNMT1** | B8 | 25,20 | 26,37 | 25,95 | 23,56 | 28,55 |
| **EGF** | B9 | 29,48 | 36,38 | 36,82 | 27,35 | Undetermined |
| **ENG** | B10 | 27,45 | 32,97 | 29,65 | 25,78 | 32,46 |
| **EPCAM** | B11 | 31,89 | 25,24 | 25,79 | 21,91 | 29,04 |
| **ERB2** | B12 | 33,82 | 32,46 | 26,95 | 22,36 | 29,67 |
| **ETFA** | C1 | 23,57 | 23,61 | 24,69 | 22,00 | 24,81 |
| **FGFR2** | C2 | 27,67 | 28,16 | 28,97 | 23,92 | 28,40 |
| **FLOT2** | C3 | 26,74 | 28,25 | 28,32 | 22,82 | 29,73 |
| **FOXA2** | C4 | 36,33 | 27,83 | 28,74 | 24,30 | 30,89 |
| **FOXP1** | C5 | 24,87 | 23,54 | 23,79 | 20,51 | 25,73 |
| **FZD7** | C6 | 25,52 | 24,29 | 25,28 | 21,98 | 24,70 |
| **GATA3** | C7 | Undetermined | 36,94 | Undetermined | 29,24 | Undetermined |
| **GSK3B** | C8 | 26,71 | 29,18 | 28,83 | 23,76 | 30,55 |
| **HDAC1** | C9 | 24,37 | 23,89 | 24,17 | 20,83 | 24,65 |
| **ID1** | C10 | 27,21 | 29,43 | 31,43 | 28,11 | 34,82 |
| **IKBKB** | C11 | 25,85 | 26,73 | 26,19 | 21,81 | 28,91 |
| **IL8** | C12 | 27,31 | 29,84 | 28,40 | 25,50 | 33,58 |
| **ITGA2** | D1 | 27,31 | 26,89 | 28,28 | 24,67 | 27,65 |
| **ITGA4** | D2 | 27,34 | 29,08 | 27,61 | 26,53 | 30,82 |
| **ITGA6** | D3 | 26,33 | 27,11 | 25,37 | 23,19 | 26,44 |
| **ITGB1** | D4 | 29,26 | 30,55 | 30,83 | 25,64 | 31,60 |
| **Jag1** | D5 | 22,66 | 25,10 | 24,85 | 20,96 | Undetermined |
| **Jak2** | D6 | 27,41 | 27,58 | 27,65 | 25,50 | 28,03 |
| **KIT** | D7 | 33,24 | 32,87 | Undetermined | 31,02 | Undetermined |
| **KITlig** | D8 | 26,87 | 29,91 | 29,30 | 25,79 | 30,71 |
| **KLF17** | D9 | 33,94 | 34,17 | Undetermined | 28,51 | 33,15 |
| **KLF4** | D10 | 28,81 | 33,63 | 30,80 | 25,11 | 33,52 |
| **Lats1** | D11 | 29,54 | 29,51 | 31,76 | 25,28 | 33,81 |
| **Lin28A** | D12 | 29,71 | 30,63 | 31,56 | 27,81 | Undetermined |
| **Lin28B** | E1 | Undetermined | 32,69 | 34,35 | 25,20 | 30,53 |
| **Maml1** | E2 | 26,87 | 26,53 | 30,38 | 22,27 | 29,09 |
| **MERTK** | E3 | 29,75 | 27,24 | 28,24 | 22,64 | 29,28 |
| **MS4A1** | E4 | 34,64 | 32,77 | 34,51 | 30,75 | 34,90 |
| **Muc1** | E5 | 30,42 | 28,66 | 29,59 | 25,39 | 31,87 |
| **Myc** | E6 | 22,89 | 25,39 | 26,82 | 21,77 | 26,36 |
| **MycN** | E7 | 28,23 | 33,70 | 32,68 | 30,47 | 34,40 |
| **Nanog** | E8 | 31,91 | 26,87 | 28,88 | 24,47 | 30,67 |
| **NFKB1** | E9 | 29,27 | 29,34 | 30,19 | 26,18 | 33,43 |
| **Nos2** | E10 | 26,48 | 33,21 | 31,24 | 27,46 | Undetermined |
| **Notch1** | E11 | 29,89 | 31,85 | 31,36 | 26,88 | 35,74 |
| **Notch2** | E12 | 23,94 | 25,31 | 25,64 | 21,59 | 25,35 |
| **Pecam1** | F1 | Undetermined | 36,91 | 36,55 | 33,02 | 36,27 |
| **Plat** | F2 | 36,35 | Undetermined | 37,43 | 38,03 | 36,82 |
| **Plaur** | F3 | 26,74 | 25,62 | 25,51 | 21,43 | 27,88 |
| **Pousf1** | F4 | 29,82 | 30,30 | 31,21 | 26,28 | 32,68 |
| **Prom1** | F5 | 26,64 | 30,56 | 31,50 | 31,70 | Undetermined |
| **PTCH1** | F6 | 33,35 | 34,45 | Undetermined | 28,79 | Undetermined |
| **PTPRC** | F7 | 30,29 | 31,31 | 34,57 | 28,84 | 34,63 |
| **sav1** | F8 | 29,60 | 28,64 | 29,04 | 24,39 | 30,11 |
| **Sirt1** | F9 | 27,54 | 26,61 | 27,32 | 23,63 | 27,43 |
| **Smo** | F10 | 28,23 | 28,45 | 28,14 | 23,72 | 29,43 |
| **Snail1** | F11 | 35,00 | 36,13 | Undetermined | 33,94 | Undetermined |
| **Sox2** | F12 | 24,87 | 27,75 | 28,61 | 24,46 | 30,15 |
| **Stat3** | G1 | 26,71 | 27,30 | 26,89 | 22,48 | 28,27 |
| **TAZ** | G2 | 27,47 | 27,72 | 27,77 | 23,15 | 30,79 |
| **TGFBR1** | G3 | 24,18 | 25,63 | 24,47 | 22,26 | 25,65 |
| **THy1** | G4 | 27,04 | 30,21 | 28,19 | 26,90 | 31,01 |
| **Twist1** | G5 | 32,04 | Undetermined | 33,58 | 27,26 | 37,39 |
| **Twist2** | G6 | 36,98 | Undetermined | Undetermined | 36,43 | 38,46 |
| **WEE1** | G7 | 27,63 | 28,18 | 29,17 | 24,51 | 30,06 |
| **WNT1** | G8 | 32,54 | 32,14 | Undetermined | 29,70 | 32,87 |
| **WWC1** | G9 | 32,76 | 33,99 | 35,04 | 30,01 | 34,30 |
| **Yap** | G10 | 27,43 | 27,53 | 29,00 | 22,91 | 33,43 |
| **ZEB1** | G11 | 23,02 | 26,44 | 26,44 | 22,94 | 25,38 |
| **ZEB2** | G12 | 26,27 | 28,16 | 28,27 | 24,50 | 28,35 |
| **Actina B** | H1 | 26,32 | 27,42 | 27,19 | 21,61 | 29,37 |
| **B2M** | H2 | 22,53 | 23,95 | 24,29 | 21,28 | 25,64 |
| **GAPDH** | H3 | 22,85 | 27,47 | 27,54 | 22,81 | 29,36 |
| **HPRT** | H4 | 26,57 | 27,37 | 27,04 | 25,59 | 28,52 |
| **RPLP0** | H5 | 23,96 | 25,17 | 26,26 | 22,12 | 28,10 |
| **HGDC** | H6 | 31,35 | Undetermined | Undetermined | 34,32 | 32,89 |
| **RTC** | H7 | 19,48 | 19,73 | 18,90 | 19,32 | 18,78 |
| **RTC** | H8 | 19,56 | 19,89 | 19,16 | 19,40 | 18,87 |
| **RTC** | H9 | 19,45 | 19,64 | 18,97 | 19,88 | 18,66 |
| **PPC** | H10 | 17,91 | 18,65 | 18,28 | 18,57 | 18,30 |
| **PPC** | H11 | 17,82 | 18,83 | 18,57 | 18,57 | 18,39 |
| **PPC** | H12 | 19,59 | 19,16 | 19,24 | 19,35 | 18,64 |
